# Gene Expression Monotonicity across Bladder Cancer Stages Informs on the Molecular Pathogenesis and Identifies a Prognostic Eight-Gene Signature

**DOI:** 10.3390/cancers14102542

**Published:** 2022-05-21

**Authors:** Rafael Stroggilos, Maria Frantzi, Jerome Zoidakis, Marika Mokou, Napoleon Moulavasilis, Emmanouil Mavrogeorgis, Anna Melidi, Manousos Makridakis, Konstantinos Stravodimos, Maria G. Roubelakis, Harald Mischak, Antonia Vlahou

**Affiliations:** 1 Systems Biology Center, Biomedical Research Foundation of the Academy of Athens, Soranou Efessiou 4, 11527 Athens, Greece; rstrog@bioacademy.gr (R.S.); izoidakis@bioacademy.gr (J.Z.); mavrogeorgis@mosaiques.de (E.M.); annamelidi@gmail.com (A.M.); mmakrid@bioacademy.gr (M.M.); 2 Mosaiques Diagnostics GmbH, 30659 Hannover, Germany; frantzi@mosaiques.de (M.F.); mokou@mosaiques-diagnostics.com (M.M.); mischak@mosaiques.de (H.M.); 3 1st Department of Urology, Laiko Hospital, National and Kapodistrian University of Athens, 11527 Athens, Greece; napomoul@hotmail.com (N.M.); kstravd@med.uoa.gr (K.S.); 4 Laboratory of Biology, School of Medicine, National and Kapodistrian University of Athens, 11527 Athens, Greece; roubel@med.uoa.gr; 5 Cell and Gene Therapy Laboratory, Biomedical Research Foundation of the Academy of Athens, Soranou Efessiou 4, 11527 Athens, Greece

**Keywords:** bladder cancer stage, transcriptomics, meta-analysis, molecular alterations, prognostic signature, urothelial carcinoma

## Abstract

**Simple Summary:**

Gene expression monotonicity is an important feature in the evolution and progression of cancer, yet has not been investigated in bladder cancer (BLCA). Most of the transcriptomic stage investigations of BLCA are limited either to a subset of stages, or to a small number of samples. Here, we leverage publicly available data to create a meta-dataset of 1135 primary BLCA transcriptomes, to identify genes and processes with a monotonal change related to higher clinical or pathologic stages. Our analysis aims to deepen the current understanding of the disease’s molecular pathogenesis, as well as to propose a prognostic gene signature based on the trait of monotonicity. Results demonstrate tumor dependencies on specific cell-cycle and metabolic microprocesses, and highlight an eight-gene signature capable of prognosing 5-year outcomes in both the discovery and validation sets.

**Abstract:**

Despite advancements in molecular classification, tumor stage and grade still remain the most relevant prognosticators used by clinicians to decide on patient management. Here, we leverage publicly available data to characterize bladder cancer (BLCA)’s stage biology based on increased sample sizes, identify potential therapeutic targets, and extract putative biomarkers. A total of 1135 primary BLCA transcriptomes from 12 microarray studies were compiled in a meta-cohort and analyzed for monotonal alterations in pathway activities, gene expression, and co-expression patterns with increasing stage (Ta–T1–T2–T3–T4), starting from the non-malignant tumor-adjacent urothelium. The TCGA-2017 and IMvigor-210 RNA-Seq data were used to validate our findings. Wnt, MTORC1 signaling, and MYC activity were monotonically increased with increasing stage, while an opposite trend was detected for the catabolism of fatty acids, circadian clock genes, and the metabolism of heme. Co-expression network analysis highlighted stage- and cell-type-specific genes of potentially synergistic therapeutic value. An eight-gene signature, consisting of the genes *AKAP7*, *ANLN*, *CBX7*, *CDC14B*, *ENO1*, *GTPBP4*, *MED19*, and *ZFP2*, had independent prognostic value in both the discovery and validation sets. This novel eight-gene signature may increase the granularity of current risk-to-progression estimators.

## 1. Introduction

Bladder cancer (BLCA) accounts for approximately 200,000 annual deaths worldwide, and had an estimated number of 570,000 new cases in 2020 [1]. It comprises a spectrum of diseases including variably recurrent low- and high-risk non-muscle-invasive (NMI) tumors, along with muscle-invasive (MI) cases characterized by poor prognosis. The disease is diagnosed pathologically according to a well-established protocol—including cystoscopy, microscopic examination of the transurethral resection of the bladder tumor material (TURBT), and urine cytology—to determine the grade of malignancy [2]. Based on the spread of the tumor within the organ, BLCA is staged as Ta when the tumor is confined within the urothelium, T1 when it spreads to the lamina propria, T2 when it invades the detrusor muscle, T3 when it grows to the peri-vesical tissue, and T4 when it spreads to the surrounding or distant tissues and organs [3]. Advances in diagnostic tools (mostly imaging technologies) and drug discovery over the years have improved patient survival and quality of life [1]. However, disease monitoring and treatment selection still rely on clinical and histological features, which is not optimal. This is well reflected in the highly heterogeneous 5-year recurrence rates (35–76%) in NMI tumors, and in the poor 5-year survival of MI and metastatic cases [4]. The suboptimal predictability of tumor recurrence leads to frequent surveillance revisits, making BLCA the most expensive malignancy to treat over the lifetime of patients [5]. An estimated 1/4 patients diagnosed with BLCA experiences financial toxicity—a term describing the mental and emotional stress related to the burden of unaffordable cancer care, which negatively affects quality of life [5,6].

In an effort to replace or enhance cytological findings, several studies have reported molecular biomarkers capable of diagnosing primary or recurrent disease in the urine samples of patients [7]. However, reliably detecting those BLCA patients who are at true risk of recurrence or progression remains an unmet challenge. Additional studies have investigated tumor tissue, which is thought to be better suited for prognosis. In a large multicenter cohort of NMI patients—particularly among those patients who had been classified as high-risk based on the traditional EORTC score table—patients with a high 12-gene expression score experienced significantly shorter progression-free survival time than those with low scores [8]. A number of studies investigating MIBC prognosis in the TCGA data on BLCA tissue have recently been published, all demonstrating the significant predictive value of molecular (omics) information from tissue [9,10,11,12,13,14].

Bladder cancer is strongly linked to environmental factors such as smoking or occupational exposure to DNA-modifying compounds [15]. At the genomic level, normal cells adjacent to dysplastic regions manifest a number of alterations on genes implicated in carcinogenesis. These are the forerunner genes (FR genes), and are often mutated or epigenetically silenced in wide areas beyond the tumor initiation site [16]. According to the combined action of field effects and clonal expansion, due to the variability in the affected FR genes among neighboring cells, some cells can acquire small advantages in growth and survival. If obtained by the progenitor cells, these properties are propagated to descendant cells, and any additional cumulative genomic abnormalities in the descendants can potentially lead to tumor initiation [17]. 

In BLCA, tumor initiation is believed to take place in the basal layer of the urothelium, where stem cells reside [18]. Increasing evidence suggests that there are at least two sets of altered FR genes, giving rise to dominant clones with distinct biology: the luminal and the basal types. Luminal tumors correlate with hyperactivation of the FGFR3 and AKT/PI3K/mTOR pathways, while basal tumors present with losses or inactivation of tumor-suppressor genes, such as *RB1*, *TP53*, and *PTEN* [4]. However, common genomic abnormalities also exist between these two entities, including mutations on chromatin-modifying genes as well as the loss of the 9q locus—events linked to the pro-tumorigenic effect of the environmental chemicals ending up in the bladder [18]. A growing body of transcriptomic investigations of the biology of luminal and basal tumors has uncovered further molecular phenotypes [19], adding to our understanding of the disease. However, due to intratumor heterogeneity, different areas within the same tumor may present with different molecular phenotypes [20], which may complicate their clinical utility. 

For the study reported here, we compiled a discovery meta-cohort of 1135 BLCA microarray transcriptomes along with two RNA-Seq validation sets, and addressed the disease as a molecular continuum of alterations. Using the stage as a checkpoint variable that reflects the cumulative processes of tumor progression, we investigated how molecular processes and gene expression levels change, starting from the non-malignant adjacent urothelium (NAU), and continuing through the disease stages Ta, T1, T2, T3, and T4. Our analysis aims to enrich the current understanding of the molecular pathogenesis of BLCA, uncovering pathways whose activation progressively increases or diminishes with cancer growth, while also reporting for the first time on the gene co-expression profiles of the disease stages. Based on the hypothesis that the expression levels of the most optimal prognostic biomarkers should follow a monotonal trend with higher disease stage, we propose an eight-gene signature with the potential of prognosing 5-year survival outcomes.

## 2. Materials and Methods

**Figure 1:**
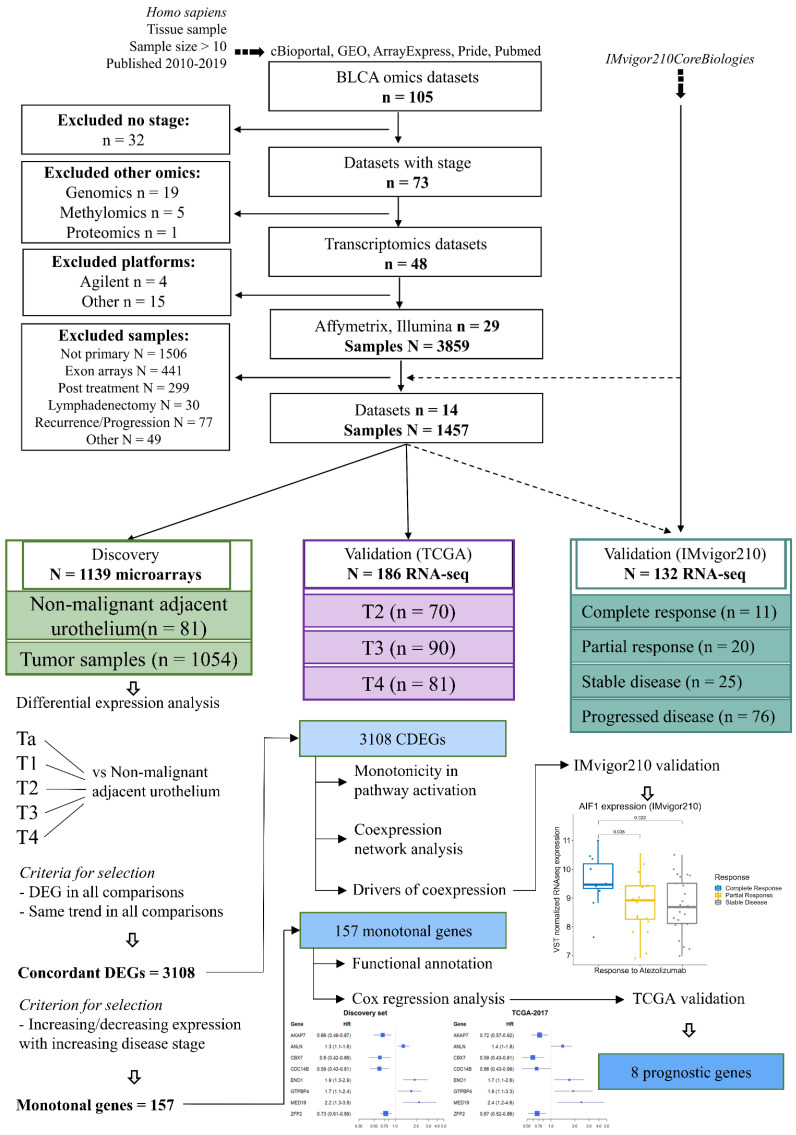
Study design and workflow for the analysis of the selected primary BLCA transcriptomes.

A comprehensive data mining strategy was employed to retrieve studies applying -omics technologies in BLCA. The overall workflow is summarized in Figure 1, and described in detail in Appendix A. This analysis focused on primary, treatment-naïve tumor transcriptomes. The selection of datasets and samples eligible for the analysis, as well as the processing and integration of raw microarray data, is detailed in Appendix A. Briefly, we compiled a microarray discovery cohort from 12 well-characterized GEO datasets (GSE121711, GSE93527, E-MTAB-1940, GSE31684, GSE104922, GSE128959, GSE83586, GSE48276, GSE52219, GSE69795, GSE13507, and GSE48075). These data (summarized in Figure 1), comprised 1054 primary bladder cancer tumor transcriptomes of treatment-naïve patients without any prior cancer history, along with profiles from 81 non-malignant urothelium tissues adjacent to the tumor site (NAU), corresponding to a total of 1135 gene expression profiles. Stage distribution among the utilized datasets is shown in Table A1. Two RNA-Seq datasets were used for validation (TCGA2017 and IMvigor210), as per availability. Table 1 shows sample allocation to the clinical variables for both the discovery and validation sets. Figure 1 illustrates the overall study design and workflow. In the discovery set, the ratio of men:women was 3.5:1, with equal distribution between NMI and MI disease (*p* = 0.99), and similar mean age at baseline diagnosis (68 years, *p* = 0.81). Percentages of NAU, NMI, and MI in the dataset were 7.1%, 43.5%, and 49.4%, respectively, with the grade distribution being as follows: 16.5% low-grade and 48.8% high-grade disease, with the remaining samples lacking available grade information. Detailed histological records were missing for 71.5% of the cohort, with the most frequently reported histology among the available records being urothelial/papillary (23.3%), and squamous differentiation being the most frequent variant (1.3%).

Gene expression across the 12 GEO datasets was harmonized using the empirical Bayes approach of the ComBat algorithm. Batch effect removal was assessed with the algorithm BatchQC (Figures S1 and S2) [21], with relative log expression and principal component analysis plots (Figure S3), with comparative analysis of expression levels between housekeeping and non-housekeeping genes (Figure S4), or using a set of 11 positive BLCA markers with known regulation across non-malignant–NMI–MI or non-malignant–low-grade–high-grade disease (Figure S5). ComBat successfully eliminated batch effects (Figures S1–S3), while maintaining the biological variability (Figures S3 and S4), BatchQC reports indicated that the variability in the corrected data was explained by stage rather than by the batch variable (Figure S2), allowing for an in-depth downstream analysis. The ComBat corrected expression matrix along with the sample clinical data are available as File S1 and File S2, respectively.

To identify genes that form a continuum of changes across BLCA stages, each of the 5 disease stages was initially compared against non-malignant adjacent urothelium (NAU). A total of 3018 genes differed significantly (Mann–Whitney *p* < 0.05), while having the same orientation of change in all comparisons. We refer to this set of genes (*n* = 3108) as concordantly differentially expressed genes (CDEGs). CDEGs were utilized to infer pathway activation scores, and to create stage co-expression networks. Pathway activation scores per sample were calculated with the ssGSEA–GSVA method [22], using the molecular signature database libraries of Hallmark, Canonical Pathways (Reactome subset), C3 (GO biological processes subset), and C5 (GTRD subset of transcription factor targets). Dorothea (https://github.com/saezlab/dorothea, accessed on 16 March 2021) [23] was utilized to assess regulon activity. To further identify the subset of pathways whose activation had monotonal traits across non-malignant adjacent urothelium (NAU) and disease stages, each pathway’s activation scores across stages were compared to NAU with Mann–Whitney tests, and the direction of change was defined based on fold change (= mean of stage − mean of NAU). Monotonicity for a pathway was defined as being significantly different in all stage comparisons to NAU, and also having a continuously larger/smaller fold change with increasing stage. Stromal infiltration scores were imputed using the ESTIMATE algorithm [24].

Gene-pair co-expression weights among the 3108 CEDEGs were approximated with ensemble learning, using GENIE3 [25], while the direction of co-expression (positive/negative) was determined using Spearman’s coefficient. Out of the 3108 × 3108 = 9,659,664 gene-pair weights calculated individually per condition (i.e., NAU and 5 BLCA stages), gene-pairs with the highest GENIE3 weights, which were also positively correlated based on the Spearman’s coefficient, were used to construct networks. The cutoff for this selection was determined based on the gene size of the resulting networks; to avoid network saturation, we opted to keep their gene sizes close to half the number of CDEGS (= 1554 genes), which resulted in setting the cutoff to the top 5600 gene-pairs. Networks were constructed with igraph and were analyzed with Louvain clustering [26] to identify local modules of co-expression relationships (communities) per condition. The top five in size (= number of genes) of the communities of co-expressed genes per condition were analyzed for Gene Ontology Biological Processes with clusterProfiler [27]. Potential drug targets in the co-expression networks were defined based on the betweenness centrality metric [28], using default cutoffs (computed with igraph).

We utilized CDEGs to extract genes whose expression levels was monotonically increasing or decreasing with higher stage. Monotonicity for a gene was defined as being a CDEG and additionally having a continuously larger/smaller fold change with increasing stage (fold change, as defined in each disease stage versus NAU). Functional annotation and enrichment were performed using PubMed and the online tool GeneCards (https://ga.genecards.org/, accessed on 22 March 2021), respectively. Out of the monotonal subset, 43 genes were found to be of prognostic value (Cox univariate association with 5-year outcome). Eight of these genes, validated in the TCGA-BLCA dataset, were further utilized to construct a sample-wise scoring system—the 8-gene prognostic signature—by summing the expression values of the upregulated genes (*n* = 4) while subtracting the downregulated genes (*n* = 4). Before calculations, each of the gene expression values per sample was divided by the gene’s variation across the dataset, in order to minimize the effect of individual gene variability on the final signature score:

S_i_ = A_i_/VarA + B_i_/VarB + C_i_/VarC + D_i_/VarD − E_i_/VarE − F_i_/VarF − G_i_/VarG − H_i_/VarH (1)

where S_i_ denotes the sample-wise derived signature score, A_i_ B_i_, C_i_, D_i_, and E_i_, F_i_, G_i_, H_i_, denote the sample-wise gene expression of the 4 upregulated and 4 downregulated genes, respectively, and Var denotes the gene expression variance across the entire set of samples.

The 8-gene signature and the disease stage were further used as inputs in a multivariate Cox regression model, to identify whether they had independent prognostic value. This procedure was applied on both the discovery meta-cohort and the TCGA validation data.

Since our procedure for the detection of monotonal traits in genes and pathway activities involved multiple filtering criteria, to avoid over-elimination due to Type-II error, significance was defined at an unadjusted Mann–Whitney *p* < 0.05. In contrast, significance for pathway over-representation (clusterProfiler output) was determined by FDR correction at *p* < 0.05. Categorical variables were investigated for significance with Pearson’s chi-squared test, and were adjusted for multiple hypotheses (with the package RVAideMemoire). All reported correlation scores corresponded to Spearman’s rank coefficient. Cox proportional hazards regression was performed with the packages survminer and survival, and statistical significance was determined via the log-rank method. CIBERSORT analysis was conducted on the web platform https://cibersort.stanford.edu/, (accessed on 9 April 2021) and only samples with successful deconvolution (*p* < 0.05, *n* = 350 samples) were further used for the statistical comparisons of relative immune populations between stages. Read counts from the IMvigor data were acquired from the IMvigor210CoreBiologies, and were normalized with the variance stabilization transformation [29]. Unless stated otherwise, all processing, analyses, and visualizations were conducted in the R statistical environment (version 4.0.2).

## 3. Results

### 3.1. Pathway Activation and Gene Co-Expression Link the Cell Cycle to ECM and Metabolic Alterations

**Figure 2:**
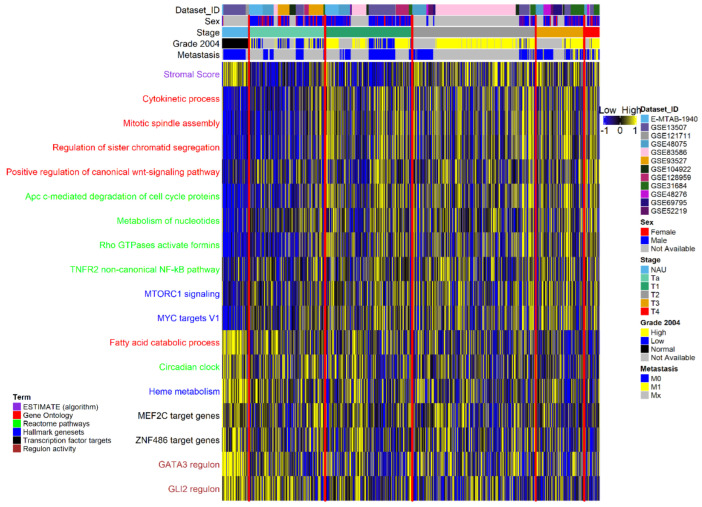
Excerpt of the pathways showing a monotonal increase or decline in their activation scores with higher stage. Pathway activation scores were z-scaled across samples for visualization. Pathways are colored based on their database of origin. The top side of the heatmap presents dataset and clinical information. Samples (columns) are ordered based on the stage variable; from left to right: non-malignant adjacent urothelium (NAU), Ta, T1, T2, T3, and T4. Red lines indicate boundaries between adjacent stages.

For initial assessment of the gene expression relationships between the non-malignant adjacent urothelium (referred to as NAU) and cancerous samples, we performed differential expression analysis of NAU versus NMI and NAU versus MI samples. When compared to NAU, a strong positive correlation (r = 0.83, *p* < 2.1 × 10^−16^) was observed between the two sets of log fold change values, suggesting concordance in abundance changes and directionality between NMI and MI tumors. To investigate transcriptional changes associated with increasing malignancy, we compiled genes being differentially expressed in all stages compared to NAU, also showing a persistent change—either up or down—and we further denoted them as concordantly differentially expressed genes (defined in the Materials and Methods Section; CDEGs, *n* = 3108). Due to their consistent regulation compared to NAU, CDEGs likely reflect fundamental alterations occurring during carcinogenesis; thus, we focused our analysis on this particular set of genes. We initially performed a ssGSEA–GSVA analysis using CDEGs, aiming to identify pathways and biological processes whose activation is continually enhanced or diminished throughout the disease stages. With increasing disease stage, our results indicated gradually stronger activation of several mitotic processes, positive regulation of the canonical Wnt pathway, mTORC1 signaling, expression of *MYC* targets, degradation of anaphase inhibitors, metabolism of nucleotides, mobility of formins, and the TNFR2/non-canonical NF-κB pathway (Figure 2, Table S1). Conversely, diminished activity was recorded for the lipid and fatty acid catabolic processes, for the metabolism of heme and, interestingly, for the circadian clock process (Figure 2). Regulon activity per sample was additionally estimated, and respective scores between disease stages and normal tissue were compared. This analysis highlighted the GATA3 and GLI2 regulons, whose activity was significantly diminished with increasing malignancy (Figure 2).

**Figure 3:**
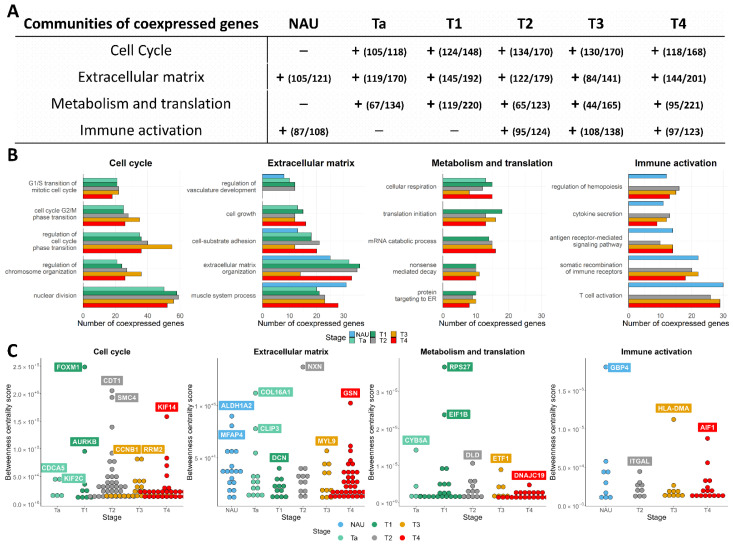
Biological process analysis of the largest (in size) co-expressed communities identified in each BLCA stage network: (**A**) Coherent communities identified and characterized across the non-malignant adjacent urothelium (NAU) and disease stages. The presence of a community is indicated by the + symbol. Numbers in parentheses show the fraction of genes with GO Biological Process annotation relevant to the community, with respect to the total number of genes found to be co-expressed in the community. (**B**) Bar plots of the most significantly enriched biological processes per community, depicting the number of co-expressed genes for each. (**C**) Hub genes identified across the studied conditions based on the betweenness centrality scores (*y*-axis).

To further investigate co-expression alterations occurring in the disease stages, an integrated network–pathway analysis was performed. Stage-specific networks were constructed and clustered to identify communities (subnetworks) of co-expressed genes (described in methods). We analyzed the five largest communities (based on the number of genes) per disease stage and NAU, and used the Gene Ontology—Biological Process (GO–BP) library to identify affected molecular processes (Figure 3, Tables S2–S7). The analysis revealed large differences in gene co-expression between NAU and disease stages, with four out of the five largest communities clearly associated with specific biological processes. 

Three out of the top five communities were consistently detected in all BLCA stage networks. Based on examination of their enriched processes, these were labeled as (1) the cell-cycle community, (2) the ECM and developmental community, and (3) the metabolic and translational community (Figure 3A,B).

**Figure 4:**
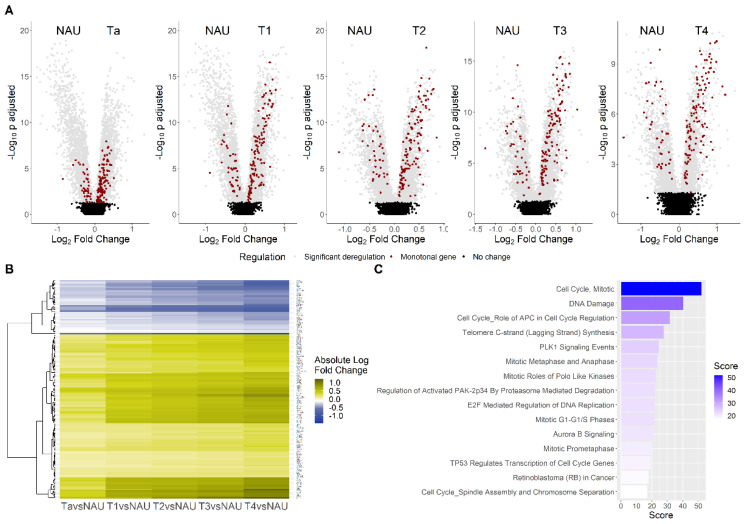
Differential expression analysis between non-malignant adjacent urothelium (NAU) and BLCA stages: (**A**) Volcano plots of the five stages’ comparisons to NAU, with the color red indicating the 157 genes showing a monotonal trend of expression across stages. (**B**) Heatmap of the absolute fold changes of the 157 monotonal genes, which were continuously either up- (yellow) or downregulated (blue), in the comparisons between disease stages and NAU. (**C**) Top 15 pathways of the 157 monotonal genes, sorted by their GeneCards enrichment score.

The cell-cycle communities of the different tumor stages involved a total of 288 genes, 178 of which had a proliferation related GO–BP annotation. Hypergeometric tests for each of the stage networks indicated highly similar cell-cycle BPs being over-represented across stages. An excerpt of the statistically most significant BPs, along with the number of implicated genes, is presented in Figure 4B. Out of the 178 cell-cycle genes, 80 were co-expressed consistently in all stage networks. These were also upregulated in tumors compared to NAU, possibly forming the backbone of cell proliferation in BLCA (Table S8). The gene size of this community increased with increasing disease stage (Ta *n* = 118, T1 *n* = 148, and MIBC *n* = 168–170 genes). The communities also included genes lacking cell-cycle GO annotation (11.9% for Ta, 17.6% for T1, 21.9% for T2, 24.9% for T3, and 29.8% for T4). An over-representation test of the 110 genes lacking GO–BP annotation across the stages revealed that 20 of them participate in the metabolism of nucleotides (unadjusted *p* = 0.03), likely suggesting a rewiring component controlling both the regulation of proliferation and the processing of nucleotides. To detect the most relevant potential drug targets within the cell-cycle communities, we determined their betweenness centrality scores. The most prominent genes included *CDC5*, *KIF2C*, *FOXM1*, *AURKB*, *CDT1*, *SMC4*, *CCNB1*, *RRM2*, and *KIF14* (Figure 4C).

**Figure 5:**
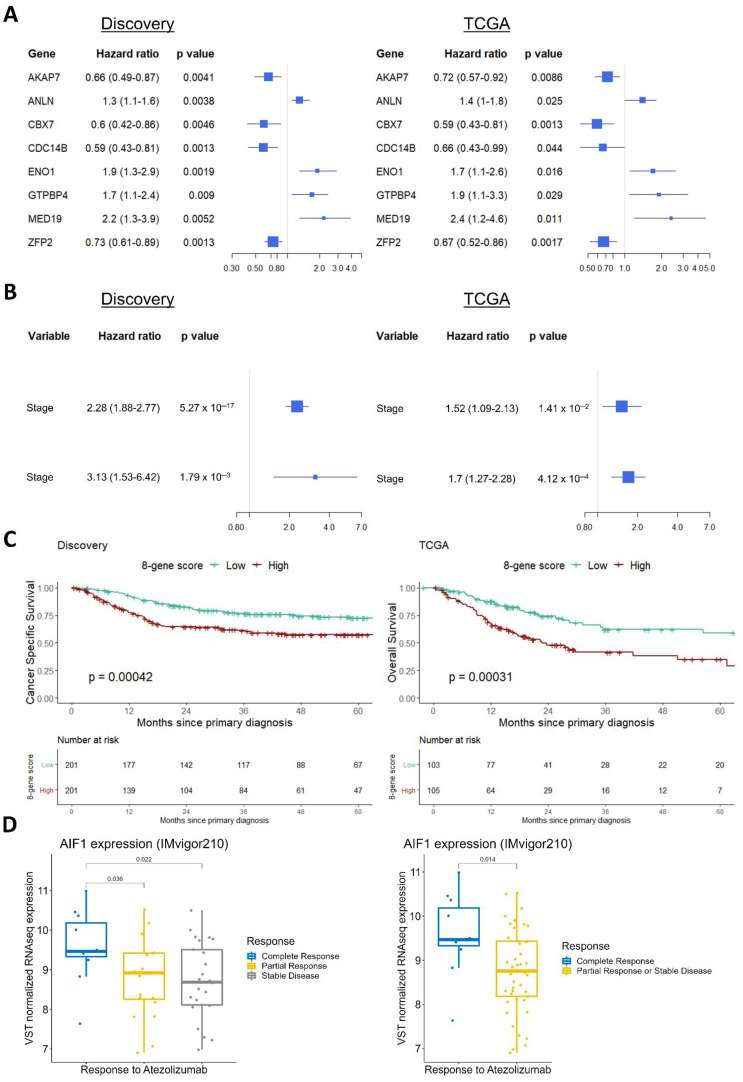
Validation of key findings in the TCGA-2017 and the IMvigor210 cohorts: (**A**) Forest plots showing hazard ratios (HRs) for the 8 monotonal genes with univariate prognostic value in both the discovery and TCGA validation datasets. (**B**) Multivariate analysis of stage and the 8-gene signature scores in the discovery and TCGA validation sets. (**C**) 5-year survival analysis between patients with high and low 8-gene signature scores, in the discovery and the TCGA data. (**D**) Data from the IMvigor210 trial illustrating *AIF1* expression across responses to atezolizumab in immunotherapy groups.

The community of ECM and developmental processes encompassed a total of 291 genes, and was enriched in cell–cell communication and cell–matrix interaction processes, in responses to microenvironmental stress, and in differentiation programs of epithelial, mesenchymal, and stem cells (Figure 5B). This GO–BP composition suggests that these co-expression signals originate either from tumoral or non-tumoral cells, or might be the product of their interaction. For example, the process of extracellular matrix organization included co-expression of 15–36 genes (depending on the stage network), of which *COL13A1*, *FGFR4*, *FOXF2*, and *SCUBE1* were co-expressed only in the NAU samples compared to the disease stages. Conversely, 26 genes, including mediators of epithelial–mesenchymal transition (i.e., *COL6A1/A2*, *COL16A1*, *MFAP5*, *MMP11*), were co-expressed in tumor tissue but not in NAU. In line with recent observations [30], we noticed that NAU presented with an active ECM remodeling profile. Sixteen of the ECM-associated genes were co-expressed both in NAU and in the NMIBC stages, including genes promoting basolateral tumor cell migration (i.e., *MMP2*, *CTSK*, *PDPN*), fibrotic collagens (i.e., *COL1A2*, *COL6A3*, *COL14A1*, *COL15A1*), and pro-angiogenic factors (i.e., *PDGFRA*, *RECK*), suggesting a pro-tumorigenic potential in the NAU. However, expression in the NAU was predicted to be driven by *ALDH1A2* and *MFAP4* (Figure 4C)—genes that are both notoriously downregulated in other genitourinary malignancies compared to normal samples [31,32], likely suggesting tumor-suppressive roles. In contrast, co-expression in the Ta stage (confined to the internal lining of the bladder) was predicted to be regulated by the hub genes *COL16A1* and *CLIP3*. *CLIP3* interacts with both *AKT1* and *AKT2* [33], and may therefore play an important role in the early AKT/PI3K/mTOR axis of hyperplastic carcinogenesis. A list containing the community’s betweenness centrality scores for each stage can be found in Table S9. 

The community of metabolism and translation encompassed mitochondrial, translational, and multiple metabolic processes being activated during carcinogenesis, and was more profound in the T1 and more advanced tumors. Cellular respiration, translational initiation, mRNA catabolic processes, nonsense-mediated decay, and protein targeting to the ER were consistently enriched in most BLCA stages. The results highlighted a set of 12 genes commonly co-expressed across stages for these processes, including *COX7B*, *DLD*, *NDUFS4*, *UQCRFS1*, *PAIP2*, *RPL15*, *RPL30*, *RPL7*, *RPS23*, *RPS27*, *RPS27A*, and *RPS4X* (Table S10). 

In addition to the abovementioned consistently detected communities in BLCA, a community enriched in processes of immune cell differentiation, cytokine secretion, and GPCR activity was identified in the NAU and the MIBC stages, and involved both innate and adaptive responses, as well as processes of immune cell adhesion and migration. This immune-associated community presented low variation in the composition of genes participating in the co-expression networks between NAU and the MIBC stages. Out of the 17 genes involved in the process of T-cell activation that were commonly co-expressed at the MIBC stages, 15 were also co-expressed in the NAU samples. To further investigate these observations, the transcriptome data per sample were deconvoluted into relative abundances of immune cell populations using CIBERSORT, and cell fractions between disease stages were compared. Significant results were obtained for the following populations: CD8+, activated CD4+, activated NK, monocytes, M2 macrophages, and activated dendritic cells (Figure S6). Our results indicated differential commitment of immune cells to NAU and the BLCA stages. NAU samples (*n* = 37) were significantly more infiltrated with CD8+ (*p* = 0.046) and monocytes (*p* = 4.6 × 10^−4^) than tumor samples (*n* = 313), consistent with an overrepresentation of the excluded over the inflamed phenotype, as previously seen in the IMvigor210 trial data [34]. However, compared to tumor samples, NAU samples had significantly less abundance of activated CD4+ cells (*p* = 5.28 × 10^−3^), macrophages (*p* = 16.8 × 10^−5^), activated dendritic cells (*p* = 0.0002), and activated NK cells (*p* = 0.015). Generally, NMIBC had lower infiltration burden than MIBC. Activated dendritic cells were significantly increased in Ta tumors (*n* = 34) compared to other BLCA stages (*p* = 0.024). The abundance of CD8+, activated NK cells, and M2 macrophages increased linearly with higher malignancy. Interestingly, *AIF1*—a gene that promotes macrophage survival and M2 polarization [35]—was predicted to be a driver of immune co-expression in the T4 tumors. Along with the CIBERSORT results, which indicate a greater abundance of M2 macrophages in the T4 samples, we hypothesize that *AIF1* is actively involved in the immune suppression and, thus, its expression levels might indicate putative candidates for immune checkpoint inhibition. A list containing the community’s betweenness centrality scores for each stage can be found in Table S11.

### 3.2. Monotonicity in Individual Genes, Prognostic Signatures, and Validation

Using the 3108 CDEGs, we extracted genes with a monotonal (i.e., continuously increasing or decreasing) change in expression in the spectrum NAU–Ta–T1–T2–T3–T4. A total of 157 genes was identified with the trait of monotonicity, of which 118 were up- and 39 were downregulated with increasing stage (Figure 4, Table S12). Functional analysis revealed that for 46 of these genes, experimental evidence on mediating cell-cycle progression exists (Table S12). Upregulated cell-cycle-associated genes (*n* = 44) were not phase-specific, and included cyclins, DNA polymerases, regulators of the cohesin complex, and kinetochore components. The list also included 23 genes involved in signal transduction (Table S12), 6 of which (*ARHGAP11A*, *AURKA*, *CDKN3*, *PBK*, *PLK1*, and *RRM2*) promote cell-cycle progression, and were all upregulated with increased stage. The data also indicated an overactivation of the Wnt pathway with increasing disease stage, with its upstream inhibitor *APCDD1* being downregulated and its activating ligand *WNT2* upregulated (Table S12). In total, 14 of the 157 genes were transcriptional or translational regulators (Table S12), including genes with known upregulation in bladder cancer (e.g., the transcription factors *E2F1* and *DEPDC1* [36,37]). Based on the monotonal changes with higher stage, increased androgen receptor activity may be predicted, as both its translational enhancer *BUD31* [38] and its downstream transcription factor *ELK1* [39] were upregulated. In total, 4 of the 157 genes (*HTR2C*, *LRP8*, *NENF*, and *NMU*) are involved in neurotransmission or neuronal development, all of which were upregulated (Table S12). Among the 157 genes, 21 were of poorly described or unknown function (Table S12), including the oncogenic factor *TRIM65* [40], which was found to be upregulated with increasing bladder cancer stage. Further functional enrichment using GeneCards for the 157 genes verified their involvement in cell-cycle pathways, with the top hits being related to the regulation of the anaphase-promoting (APC) complex (score = 31.53), to PLK1 (score = 24.47) and Aurora B (score = 20.95) signaling, and to TP53 (score = 19.06) and RB1 (score = 17.85) cell-cycle checkpoint control (Figure 4C, Table S13). Univariate Cox regression analysis indicated 48 genes with potentially prognostic impact at *p* < 0.01 (Table S14).

Due to the lack of an RNA-Seq dataset comprising the entire disease stage spectrum of BLCA incidents, the observed stage alterations in the discovery set were investigated for their reproducibility in the TCGA-BLCA RNA-Seq data [41]. To align the validation samples to the discovery set, patients with unknown history of prior treatment for non-muscle-invasive bladder cancer, as well as patients with a history of other malignancies, were excluded. Differential expression analysis between the available stage comparisons (T3 vs. T2 and T4 vs. T3) in the TCGA data validated 43 of the 157 monotonal genes (Table S15). Cox regression analysis in the TCGA data validated 8 out of the 48 monotonal genes that were found to be of prognostic value in the discovery set (Table S14), including *MED19*, *ENO1*, *ANLN*, and *GTPBP4*, higher levels of which were associated with worse survival, and *CBX7*, *ZFP2*, *AKAP7*, and *CDC14B* higher levels of which were associated with better survival probability (Figure 5A). We utilized these eight genes to construct a combined score characterizing each individual sample (see the Materials and Methods Section). Along with the disease stage, the eight-gene signature had independent prognostic value, both in the discovery and in the validation set (Figure 5B). Specifically, we constructed a survival model to compare 5-year survival rates between those with high and low eight-gene signature scores (defined by a median cutoff). Patients with a high eight-gene signature score had a worse 5-year survival probability in the discovery set, and this finding was validated in the TCGA data (Figure 5C). The eight-gene signature did not differ significantly between males and females (*p* = 0.36), and was weakly associated with age and stage (Figure S7), suggesting its independent value with respect to other clinical variables. In an attempt to verify the co-expression analysis findings, stage-specific co-expression networks were also created using the TCGA data, and were clustered using the Louvain algorithm. GO–Biological Process analysis of the communities validated the differential segregation of the cell cycle, extracellular matrix, and immune activation processes to distinct communities (Figure S8). In order to validate the value of *AIF1* as a candidate biomarker of response to immunotherapy, we analyzed RNA-Seq data from the IMvigor210 study—a trial investigating responses to atezolizumab immunotherapy in patients with metastatic BLCA. High *AIF1* expression in the IMvigor data was associated with a complete response to atezolizumab (Figure 5D).

## 4. Discussion

Integration of BLCA molecular data has been previously performed in the context of characterizing molecular subtypes [42] or validating results of either (single-cell) scRNA-Seq [43] or RNA-Seq re-analysis [44]. In this study, we performed an integrated meta-analysis of datasets from non-tumor-bearing adjacent urothelium and BLCA stages, aiming to identify continuous as well as concerted gene expression alterations with increasing malignancy. To our knowledge, this is the first attempt to associate molecular alterations with clinical classification based on the analysis of more than 1000 well-characterized primary tumor datasets. Instead of focusing on molecular subtypes, we increased the power and addressed the disease as a continuum under the assumption that individual samples reflect different snapshots of the whole process. Our novel design, based on the hypothesis of continuous evolution through the stages, was successfully applied here, and resulted in novel findings on gene regulation associated with cancer progression. 

Starting from a normalized expression dataset comprising 12 microarray cohorts, we identified genes that were differentially expressed between disease stages and NAU (CDEGs), and further analyzed them for pathways/processes that were progressively altered with higher stage, for changes in the co-expression profiles of stages, and for genes showing a monotonal change in expression with higher stage. Our analysis highlighted expected landmark pathways, such as the mTORC1 pathway [45] and *MYC* targets [46], which were upregulated, but also novel downregulated pathways, such as the circadian clock and the metabolism of heme. These results, for the first time, associate BLCA progression with the disruption of the circadian homeostasis, and with iron metabolism deficiencies—events that are thought to be tumorigenic [47,48], but their exact mechanism of action is not well understood. In addition to the GATA3 regulon—a known driver of luminal biology—we found a novel progressive downregulation of the *GLI2* regulon. GLI proteins are transcription factors of the Sonic hedgehog (Shh) pathway, and although *GLI2* expression levels are positively correlated with more invasive BLCA cell lines, Shh genes do not behave accordingly [49]. Our results validate these observations, as the entire regulon of the *GLI2* TF was inactivated with increasing BLCA malignancy, suggesting no potential therapeutic effect of its inhibition.

Tumor initiation in the bladder is thought to occur within the basal layers of the urothelium, when the accumulated burden of mutations dysregulates cells’ homeostatic mechanisms, favoring uncontrolled proliferation over apoptosis [18]. The process of initiation is lengthy in time, and affects the entire neighborhood of the adjacent cells, which are continuously exposed to a pro-tumorigenic environment. Here, we found that most of the alterations in the non-tumor-bearing adjacent cells involve genes operating during embryogenesis or during ECM remodeling (Community 3, Table S2). These are likely among the first to acquire an organized pattern of co-expression. Interestingly, co-expression in NAU tissue was driven mostly by *ALDH1A2* (and partly by *MFAP4*), which catalyzes the formation of retinoic acid (RA). In the progenitor cells, during embryonic development, receptors of RA form complexes with chromatin modifiers, leading to the activation of self-renewal and differentiation programs [50]. These data give rise to the hypothesis that the cells in the NAU may express and maintain parts of a stem-cell-like RA-related program. Indeed, the biological process of response to RA appears to be enriched in the ECM communities of NAU (*p* = 4.57 × 10^−5^) and the T2 (*p* = 1.12 × 10^−2^) stage. T2 tumors are far more de-differentiated in comparison to both NAU and NMBIC, and can host multiple genetic differentiation components [41]. 

The immune activation community was present in both the NAU and MIBC samples. The results of the CIBERSORT analysis showed that within the bladder tumors, and with increasing stage, most monocytes preferentially differentiate into macrophages with M2 polarization. This is consistent with the findings of Chen et al. [43], who analyzed scRNA-Seq data of BLCA patients, and observed a similar pattern of differentiation for monocytes. In our data, T4 samples had the highest proportion of M2 macrophages, which could partially explain their immunosuppressive state. A novel finding here is the observation that *AIF1* appears to drive co-expression in the immune cells of T4 tumors. Interestingly, high *AIF1* expression was associated with complete response to atezolizumab (Figure 5D)—a finding that may have implications for patient selection for immunotherapy. Together with the observation that *AIF1* is highly expressed in macrophages responding to M2 stimulation [51], the data suggest that *AIF1* expression in the M2 macrophages could potentially trigger a PD-L1 signature in the tumor and the surrounding immune cells, leading to immune suppression [52], but further work is required to validate these preliminary observations. 

We specifically searched for genes showing a monotonal trend in their expression level with increasing disease stage, as this property may mark those genes whose quantification could offer additive prognostic value. Out of the 157 identified monotonal genes, almost half of them were components of the cell-cycle machinery, kinases signaling positively for it, or transcription factors responsible for the expression of cell-cycle genes. In total, 8 out of the 48 monotonal genes with prognostic value in the discovery cohort were validated in independent RNA-Seq data, and were utilized to develop a sample-wise gene signature. Of these, only *ENO1* (higher levels of which had been previously linked with worse BLCA outcomes [53]) and *CBX7* (downregulation of which was associated with worse survival [54]) were described previously; the remaining six genes associated with survival are novel. *MED19*, a component of the mediator complex that regulates the transcription of RNA polymerases, was found via IHC to be overexpressed in human BLCA compared to normal tissues, and its knockdown in the T24 and 5637 bladder cell lines resulted in cell-cycle arrest at the G0/G1 checkpoint, along with attenuation of cell growth [55]. The involvement of *GTPBP4* in BLCA’s development has not been characterized, but oncogenic properties have been attributed to this gene in hepatocellular carcinoma [56]. *ANLN*, *AKAP7*, and *CDC14B* are thought to regulate bladder cell growth and apoptosis in a TP53-independent manner [57]. *ICA1L* is naturally expressed in sperm cells. Its role in BLCA has not yet been described. The *CDC14B* gene is located on the 9q chromosome—a region that is often deleted in BLCA. This might also explain its overall downregulation in malignancy in comparison to NAU, with additional mechanisms (such as the increasing number of tumor cells) resulting in the observed further downregulation with increasing stage, as observed in the discovery set. *CDC14B* is believed to dephosphorylate *TP53* [58], but the functional consequences on the mitotic or DNA damage repair pathways are not yet well clarified [59]. *CBX7* is a component of the chromatin-modifier PRC1 complex, and is required for the propagation of the transcriptionally repressive state of multiple genes through cell division during embryonic development [60], including *Hox* genes [61]. Expectedly, while *CBX7* levels monotonically decreased, we noted that *HOXC6* and *HOXC9* were both monotonically upregulated with increasing malignancy (Table S1). *ZFP2* is a probable transcription factor, and evidence suggests that it plays an epigenetic role as well [62]. High loads of mutations in *ZFP36*—another member of the ZFP family—were associated with upper tract urothelial carcinoma [63]. 

Our study has its limitations, including the retrospective nature of the analysis, and restrictions in the validation set imposed by a lack of samples from all disease stages, which prevented validation of some of our observations—particularly for NMIBC. Clinical stage assignment is known to have varying rates of error. However, the high number of samples used in each of the stage categories is expected to balance out the error to some extent, while increasing the power of the received results. It should also be noted that our scope was to identify, if present, any common “core” molecular themes with high statistical power during the evolution of BLCA. This does not rule out the existence of intra-tumoral heterogeneity, which still has to be considered (together with the observed molecular changes, as defined in our study) when predicting therapeutic response.

## 5. Conclusions

The progression of BLCA is associated with monotonal alterations both in individual genes and in molecular pathways, and these alterations can be leveraged as prognosticators, as well as for the development of novel combinatorial drug therapies. The suggested eight-gene signature may help in decision making with regards to therapeutic options and surveillance schedules. However, further validation of the signature in clinical trials is required before clinical implementation.

## Figures and Tables

**Table 1:** Clinical data of the cohorts used.

Kerrypnx	Discovery Set	TCGA-BLCA-2017	IMvigor210
**No. of patients**	1135	188	132
**Median age (range)**	67 (24–95)	69 (34–90)	
**Sex**			
Female	135 (11.9%)	45 (23.9%)	29 (22.0%)
Male	459 (40.5%)	143 (76.1%)	103 (78.0%)
No info	541 (47.6%)	0 (0.0%)	0 (0.0%)
**Stage**			
NAU	81 (7.1%)	0 (0.0%)	
Ta	229 (20.2%)	0 (0.0%)	
T1	262 (23.1%)	0 (0.0%)	
T2	371 (32.7%)	70 (37.3%)	
T3	146 (12.9%)	90 (47.9%)	
T4	46 (4.0%)	28 (14.8%)	
**Grade**			
G1	13 (1.1%)	0 (0.0%)	
G2	164 (14.5%)	0 (0.0%)	
G3	372 (32.8%)	188 (100%)	
Gx	13 (1.1%)	0 (0.0%)	
No info	573 (50.5%)	0 (0.0%)	
**Response to atezolizumab**			
Complete			11 (8.3%)
Partial			20 (15.2%)
Stable disease			25 (18.9%)
Progressed disease			76 (57.6%)

## Data Availability

This study used publicly available data. The raw microarrays used here are publicly available in the Gene Expression Omnibus under the accession codes GSE121711, GSE93527, GSE31684, GSE104922, GSE128959, GSE83586, GSE48276, GSE52219, GSE69795, GSE13507, and GSE48075, and in ArrayExpress under the accession code E-MTAB-1940. TCGA-BLCA-2017 molecular and clinical data were downloaded from cBioPortal. IMvigor molecular and clinical data were acquired through the IMvigor210CoreBiologies R package. The processed, ComBat-normalized matrix (discovery set) and the sample clinical data are available as Supplements. The codes used to generate the results and figures are available upon reasonable request.

## Supplementary Materials

The following supporting information can be downloaded at: https://www.mdpi.com/article/10.3390/cancers14102542/s1, Figure S1: Batch effect in the non-corrected data. (A) Variation explained by clinical stage and dataset ID. (B) Fraction of genes affected, by batch. (C) Heatmap of Pearson’s correlation coefficients between sample pairs. (D) First two principal components showing sample relationships; Figure S2: Batch effect in the corrected data. (A) Variation explained by clinical stage and dataset ID. (B) Fraction of genes affected by batch. (C) Heatmap of Pearson’s correlation coefficients between sample pairs. (D) First two principal components showing sample relationships; Figure S3: Gene expression and sample distribution in the corrected data. (A) Gene expression distribution (*y*-axis) among the 1135 samples (*x*-axis) in the discovery cohort, after ComBat normalization. (B) A 3D representation of the principal component analysis results, showing sample allocation in the discovery cohort after ComBat normalization; Figure S4: Mean expression and median absolute deviation of the ComBat data. Plots demonstrating preservation of the housekeeping properties in the adjusted data, i.e., higher mean expression and lower dispersion when compared to non-housekeeping genes. Left: housekeeping genes defined based on microarray analysis of several cancers. Right: housekeeping genes defined based on RNA-Seq analysis of several cancers; Figure S5: Gene expression of 11 known BLCA markers among clinical conditions in the ComBat data; Figure S6: CIBERSORT analysis of the ComBat data, showing statistically significant results on the relative proportions of immune cells between BLCA stages; Figure S7: Correlation analysis between the 8-gene signature score and the age (left) and stage of the patients (right); Figure S8: Functional annotation of the top 5 largest molecular communities of co-expressed genes detected in the T2, T3, and T4 stages of the TCGA cohort, demonstrating a significant overlap with the cell-cycle, extracellular matrix, and immunity communities of the discovery data; Table S1: Processes, pathways and regulons that follow a monotonically increasing/decreasing activation with increasing Bladder Cancer stage (NAU = Non-malignant Adjacent Urothelium); Table S2: Gene Ontology Biological Processes for the Non-malignant Adjacent Urothelium (NAU) communities of coexpressed genes; Table S3: Gene Ontology Biological Processes for the Ta stage communities of coexpressed genes; Table S4: Gene Ontology Biological Processes for the T1 stage communities of coexpressed genes; Table S5: Gene Ontology Biological Processes for the T2 stage communities of coexpressed genes; Table S6: Gene Ontology Biological Processes for the T3 stage communities of coexpressed genes; Table S7: Gene Ontology Biological Processes for the T4 stage communities of coexpressed genes; Table S8: Genes of the cell-cycle community and their betweeness centrality scores; Table S9: Genes of the ECM and developmental community and their betweeness centrality scores; Table S10: Genes of the metabolism and translation community and their betweeness centrality scores; Table S11: Genes of the immune community and their betweeness centrality scores; Table S12: Functionanl analysis of the 157 monotonically changing genes based on searches in Pubmed and GeneCards; Table S13: Functional analysis results for implicated pathways of the 157 monotonically changing genes using the GeneCards tool and database; Table S14: Univariate cox regression analysis of the 157 monotonal genes for the discovery and the TCGA validation cohorts. In green are highlighted genes who were validated with cox in the TCGA data; Table S15: Results of differential expression analysis (Mann-Whitney) between bladder cancer stages using only primary samples (70 T2, 90 T3, and 28 T4) from the TCGA Bladder Cancer 2017 cohort. Blue and red color indicate downregulation and upregulation, respectively; File S1: ComBat_expression_data; File S2: Clinical_data.

## Appendix A

### Appendix A.1. Database Search Strategy, Cohorts, and Processing

To perform a comprehensive investigation of available molecular data, we searched for bladder cancer (BLCA) omics studies in public repositories. All genomic urothelial cancer data from cBioPortal (including The Cancer Genome Atlas) were downloaded. Gene Expression Omnibus (GEO) was queried for transcriptomics, additional genomics, or protein array datasets using the search terms “bladder cancer” and “urothelial carcinoma”. We also queried ArrayExpress using the special filter “Array express data only” to obtain any additional datasets missing from GEO. All cohort data published or updated between 2010 and 2020, annotated as *Homo sapiens*, coming from tissue samples with sample size > 10, were retrieved. All used datasets were published and downloaded anonymized. This resulted in the collection of 105 datasets, comprising more than 8000 individuals (Figure 1, main text), encompassing genomics, methylomics, transcriptomics, and proteomics data, derived from a variety of technologies used in the field.

### Appendix A.2. Inclusion and Exclusion Criteria

We analyzed tumor transcriptomes and selected studies with at least clinical or pathological stage information per subject. As chemotherapy is known to significantly alter the molecular phenotype of the tumor cells, samples or datasets collected after the administration of (neo)adjuvant chemotherapy, as well as secondary or recurrent bladder cancers, were excluded. To compile a microarray discovery set while preserving as high integrity as possible, we chose microarray data quantified by the most frequently used single-color channel vendors (Affymetrix and Illumina). This resulted in a final dataset of 1135 patients sourced from 12 different studies (Table A1). We used the TCGA-BLCA-2017 and the IMvigor210 studies for validation purposes, and particularly analyzed primary BLCA samples collected prior to the administration of (neo)adjuvant chemotherapy (n samples: TCGA = 188, IMvigor = 132).

### Appendix A.3. Cohorts

Discovery (Microarray, Datasets = 12, Samples = 1135)

- GSE121711 [64]: Loras A, et al., 2019. *Cancers* (Basel): Study of the metabolome and transcriptome of bladder cancer patients, aiming to identify tissue and urinary metabolic signatures as biomarkers of BC. Contains 8 tumoral and 10 normal urothelium samples taken from regions adjacent to the tumor (NAU).
- GSE93527 [65]: Hurst CD, et al., 2017. *Cancer Cell*: Subtyping of Ta-staged non-muscle-invasive bladder cancer patients based on copy number alterations, and subsequent expression profiling of 79 samples.
- E-MTAB-1940 [66]: Biton A, et al., 2014. *Cell Reports*: Characterization of the luminal and basal muscle-invasive bladder cancer subtypes based on an independent component analysis of 86 transcriptomes.
- GSE31684 [67]: Riester M, et al., 2012. *Clinical Cancer Research*: A study for the identification and validation of high-risk bladder cancer. It included 93 samples, taken from patients undergoing radical cystectomy (RC) at the Memorial Sloan-Kettering Cancer Center (MSKCC), of which 3 were collected post-treatment and were excluded from the analysis.
- GSE104922 [68]: Therkildsen C, et al., 2018. *Molecular Oncology*: Identification of molecular profiles in 41 bladder cancer patients with Lynch syndrome.
- GSE128959 [69]: Sjödahl G, et al., 2020. *International Journal of Cancer*: An analysis of multiple samples taken from 73 patients upon recurrence/progression, investigating transcriptional alterations and subtype shifts during disease development. Expression profiles are available for 55 bladder tumors, of which 2 were excluded due to missing stage information.
- GSE83586 [70]: Sjödahl G, et al., 2017. *The Journal of Pathology*: This study utilizes tumor samples from 307 advanced bladder cancer patients in order to compare molecular classification to tumor cell phenotype (the latter assessed via immunohistochemistry). We excluded six samples due to the absence of stage information.
- GSE48276 [71]: Choi W, et al., 2014. *Cancer Cell*: The MDA molecular classification of muscle-invasive bladder cancer. The GEO dataset contains 116 expression profiles, 16 of which were collected post-treatment and, thus, excluded. An additional set of 20 profiles had no information on the timing of sample collection, and were also excluded.
- GSE52219 [71]: Choi W, et al., 2014. *Cancer Cell*: The MDA molecular classification of muscle-invasive bladder cancer. The GEO dataset contains 23 FFPE pretreatment tumors from a phase III clinical trial, which were used to validate the MDA classification.
- GSE69795 [72]: McConkey, et al., 2016. *European Urology*: Investigation of gene expression signatures predicting response to methotrexate, vinblastine, doxorubicin, and cisplatin with bevacizumab. The GEO dataset contains 61 profiles, of which 23 and 1 were excluded due to post-treatment collection and the absence of stage information, respectively.
- GSE13507 [73]: Kim WJ, et al., 2010. *Journal of Clinical Oncology*: Analysis of primary bladder cancer for the identification of a prognostic gene expression classifier. The GEO dataset contains 256 profiles, of which 9 are normal bladders, 58 are NAU, 1 profile is taken from a C57BL/6 mouse sub-strain, 165 are primary tumor samples, and 23 correspond to recurrences. Due to the low number of normal bladder samples represented in the compiled discovery cohort, we grouped them together with the NAU samples. Samples collected after recurrence (*n* = 23), as well as the expression profile of the murine strain, were excluded from analysis.
- GSE48075 [71]: Choi W, et al., 2014. *Cancer Cell*: The MDA molecular classification of muscle-invasive bladder cancer. It includes 142 primary tumors of both non-muscle-invasive and muscle-invasive bladder cancer.

Validation (RNA-Seq, Datasets 2, Samples = 318)

- TCGA-BLCA-2017 [42]: Robertson AG, et al., 2017. *Cell*: The TCGA molecular subtyping of muscle-invasive bladder cancer. Molecular and clinical data were downloaded from cBioPortal, containing 412 tumor samples. Of these, 109 had history of other malignancies, and were excluded as potentially being secondary bladder cancers. Additionally, 105 samples had unknown history of prior treatment for non-muscle-invasive bladder cancer, 2 samples were collected post-NAC, and 8 more samples had no stage information, so they were all excluded from further analysis (n analyzed = 186 samples).
- IMvigor210 [34]: Mariathasan S, et al., 2018. *Nature*: A study investigating molecular determinants of tumor response to atezolizumab immunotherapy, administered to metastatic bladder cancer patients. Transcriptomic profiles are available on GitHub (R package “IMvigor210CoreBiologies”) for 298 patients, from which we analyzed only samples collected pre-NAC (*n* = 132 samples).

**Table A1:** Stage distribution across the 12 microarray datasets used for the discovery set.

Technology	Platform	Dataset ID	NAU	Ta	T1	T2	T3	T4	Study Size
Affymetrix	GPL17586	GSE121711	10	3	2	3			18
Affymetrix	GPL17586	GSE93527		79					79
Affymetrix	GPL570	E-MTAB-1940	4	41	41				86
Affymetrix	GPL570	GSE31684		5	10	16	41	18	90
Affymetrix	GPL6244	GSE104922		19	7	10	5	0	41
Affymetrix	GPL6244	GSE128959		13	39				53
Affymetrix	GPL6244	GSE83586		13	44	241	1	1	301
Illumina	GPL14951	GSE48276			2	6	23	6	37
Illumina	GPL14951	GSE52219				15	7	1	23
Illumina	GPL14951	GSE69795			3	7	27		37
Illumina	GPL6102	GSE13507	67	23	80	31	19	12	232
Illumina	GPL6947	GSE48075		33	34	42	23	8	142
		**Stage size**	81	229	262	371	146	46	1135

### Appendix A.4. Integration and Processing of the Transcriptomes

Pretreatment, raw microarray profiles from the 12 GEO datasets were summarized to the gene level with the package oligo [74]. Affymetrix arrays were normalized via the RMA method, while Illumina arrays were filtered based on detection *p* values < 0.05, followed by quantile normalization, the addition of 1, and transformation to the natural logarithmic scale. Data were annotated using biomaRt (v2.42.1) [75], and microarray probes matching to multiple genes were excluded from downstream analysis. The probe with the highest mean across arrays was selected as representative in cases where multiple probes matched to the same gene. To completely avoid missing values, merging of expression matrices was based on the intersection of genes between datasets, using the HUGO Gene Symbol as a key. Batch effects across different studies in the discovery data were removed with ComBat [76], and the quality of the corrected data was assessed with BatchQC [21] (Figures S1 and S2), with boxplots of expression distribution per sample and principal component analysis plots of sample relationships (Figure S3), with gene expression comparisons of housekeeping genes to other genes (Figure S4) [77], and with a set of 12 positive control BLCA markers with known regulation across normal–NMI–MI or across normal–low-grade–high-grade disease (Figure S5).
