# Supplementary material for: Gene Expression Monotonicity across Bladder Cancer Stages Informs on the Molecular Pathogenesis and Identifies a Prognostic Eight-Gene Signature
Source: Cancers (Basel). 2022 May 21;14(10):2542. doi: 10.3390/cancers14102542 (PMC9140126; doi:10.3390/cancers14102542)
Supplement: Supplementary file 1 [file cancers-14-02542-s001.zip › cancers-1680775-suppl-figures.pdf]

cancers

MDPI

Article

Gene Expression Monotonicity across Bladder Cancer Stages Informs on the Molecular Pathogenesis and Identifies a Prognostic Eight-Gene Signature

Rafael Stroggilos [1], Maria Frantzi [2], Jerome Zoidakis [1], Marika Mokou [2], Napoleon Moulavasilis [3], Emmanouil Mavrogeorgis [1,†], Anna Melidi [1], Manousos Makridakis [1], Konstantinos Stravodimos [3], Maria G. Roubelakis [4,5], Harald Mischak [2] and Antonia Vlahou [1,*]

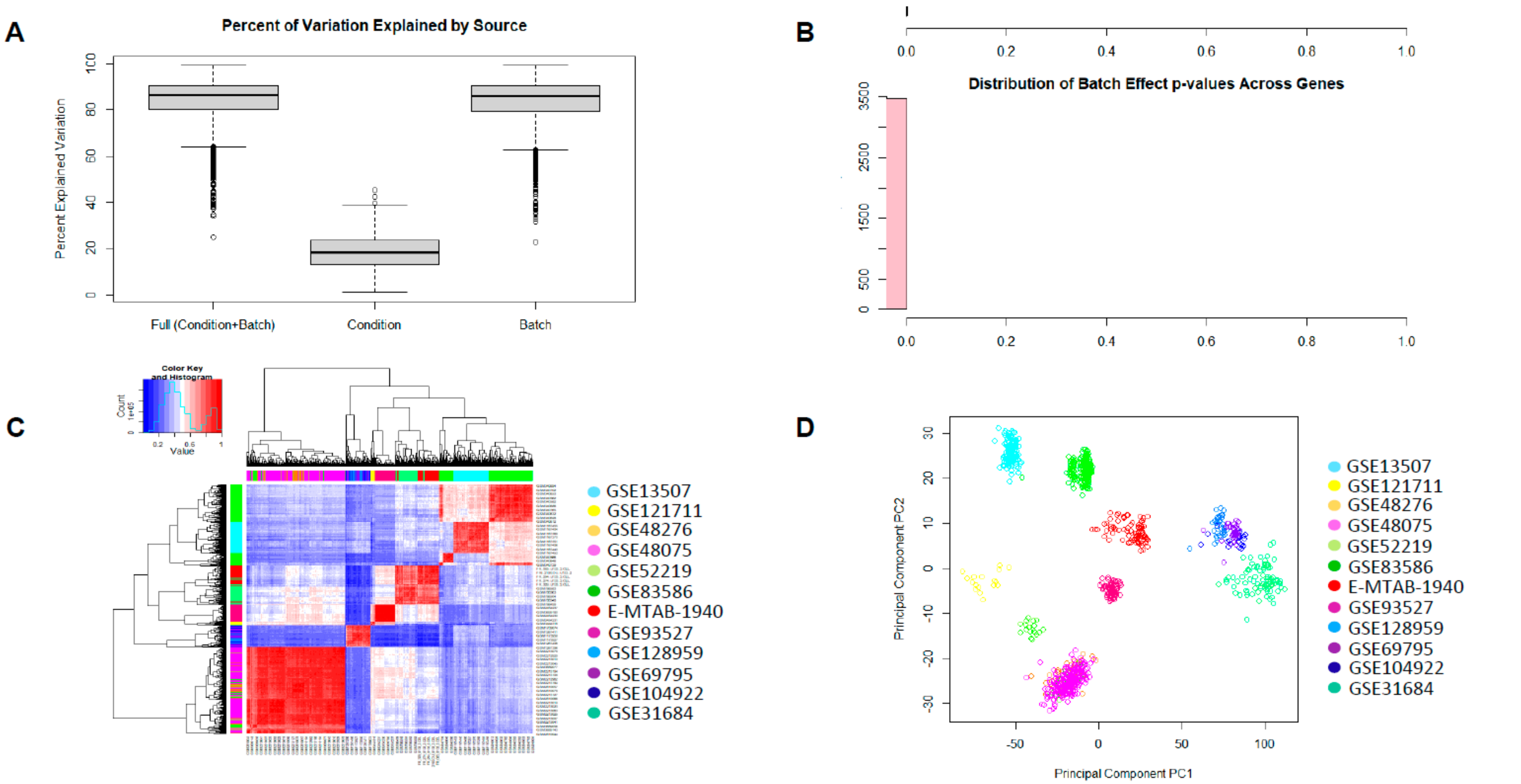

Supplemental Figure S1: Batch effect in the non corrected data (before adjusting with ComBat. A) Variation explained by clinical stage (Condition) and datasetID (Batch). B) fraction of genes affected by batch (all genes have $p < 0.05$ meaning that they are significantly affected by batch). C) Heatmap of pearson correlation coefficients between sample pairs (column and row colors correspond to the 12 datasets). D) First two principal components showing sample relationships (colors correspond to the 12 datasets).

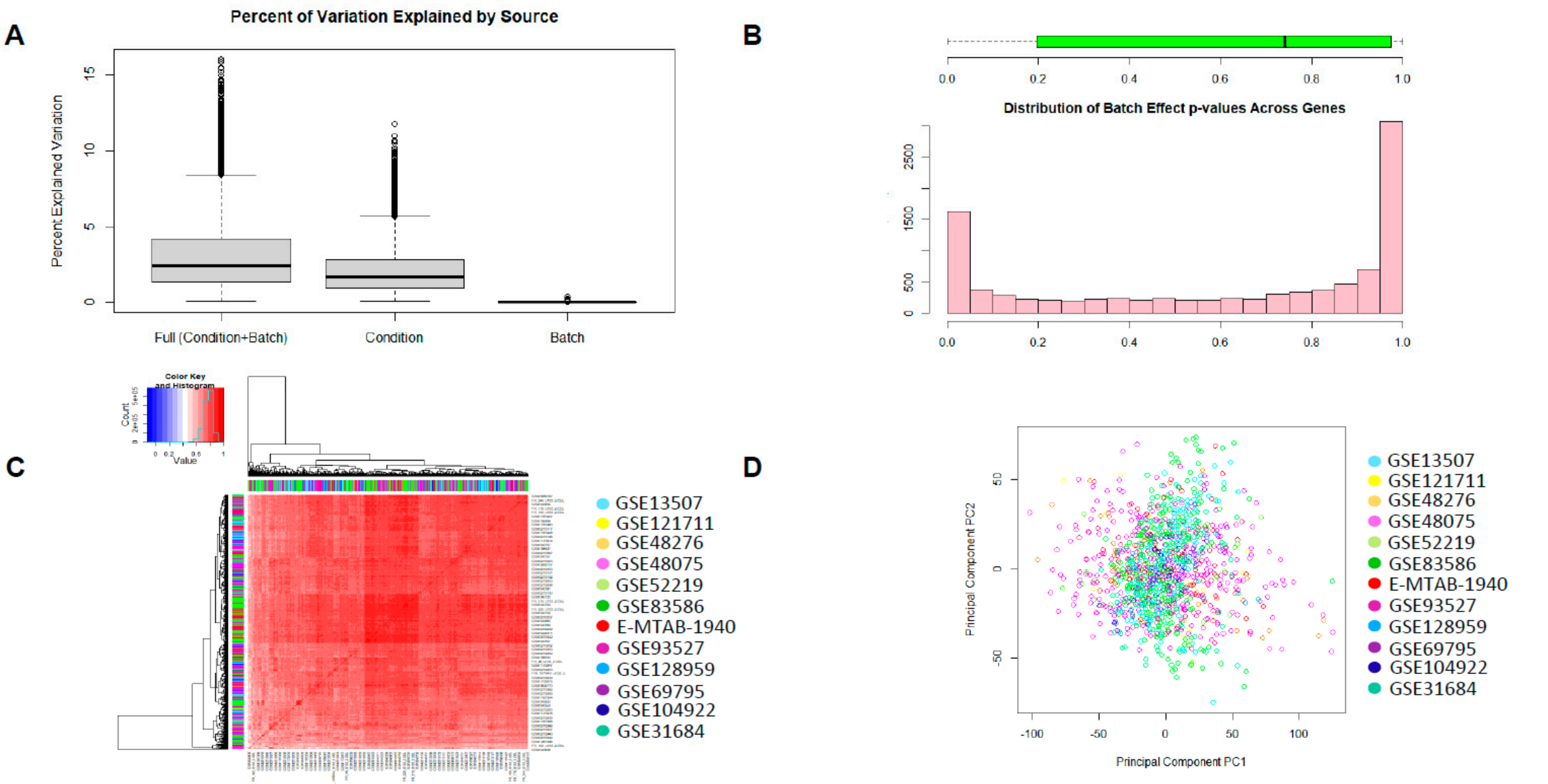

Supplemental Figure S2: Batch effect in the corrected data (after adjusting with ComBat. A) Variation explained by clinical stage (Condition) and datasetID (Batch). B) fraction of genes affected by batch (most genes have p > 0.05 meaning that they are not significantly affected by batch). C) Heatmap of pearson correlation coefficients between sample pairs (column row colors correspond to the 12 datasets). D) First two principal components showing sample relationships (colors correspond to the 12 datasets).

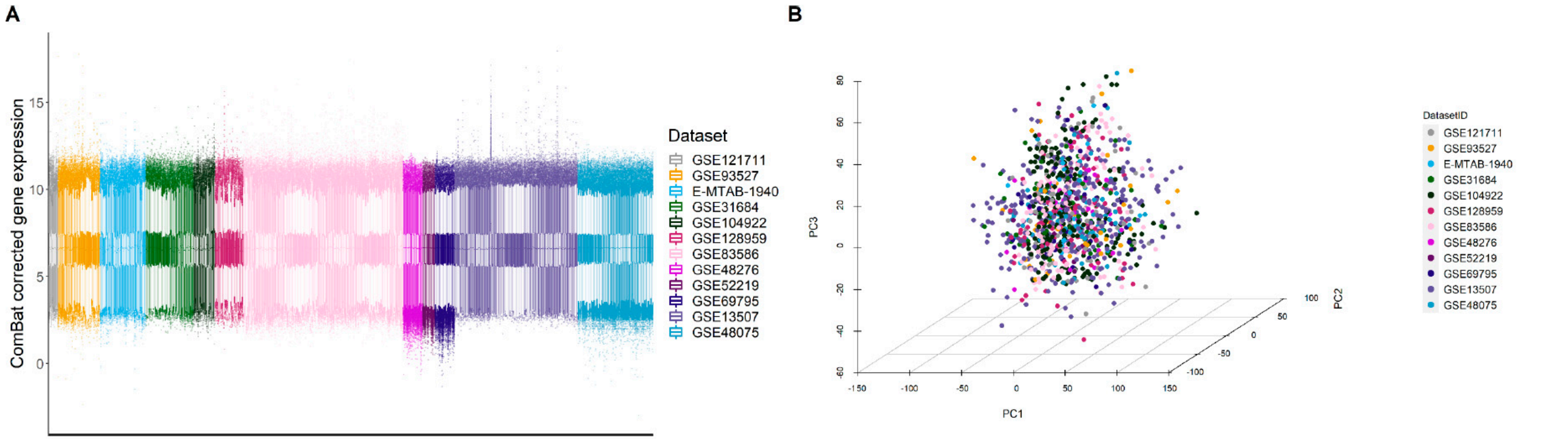

Supplemental Figure S3: Gene expression and sample distribution in the adjusted data. A) Gene expression distribution (y axis), among the 1,135 samples (x axis) in the discovery cohort, after ComBat normalization. B) 3D representation of the Principal Component Analysis result showing sample allocation in the discovery cohort, after ComBat normalization.

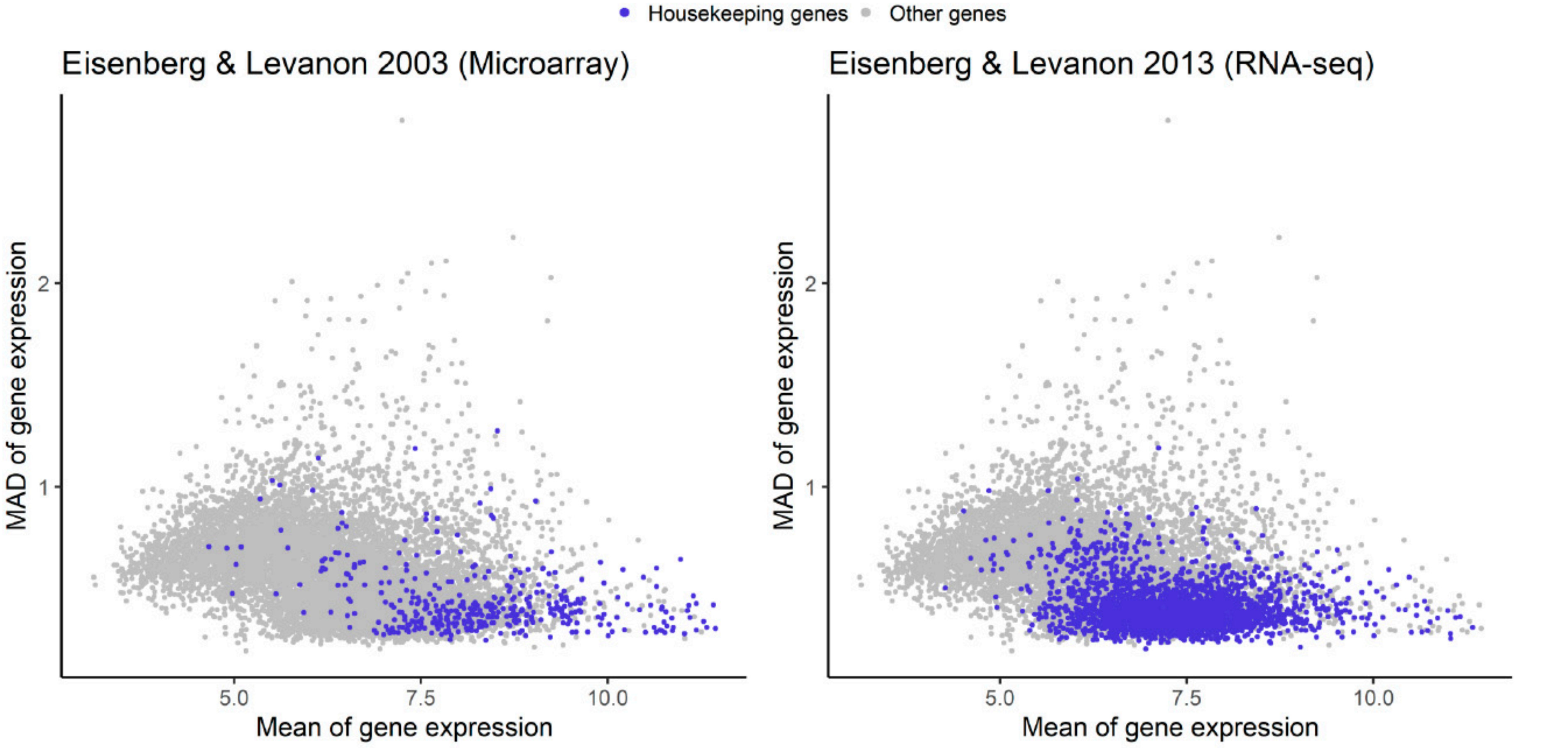

Supplemental Figure S4: Mean expression and median absolute deviation of the ComBat data. Plots demonstrating preservation of the housekeeping properties in the adjusted data. i.e. higher mean expression and lower dispersion when compared to non housekeeping genes. Left: housekeeping genes defined based on microarray analysis of several cancers, Right: housekeeping genes defined based on RNAseq analysis of several cancers. MAD: Median Absolute Deviation.

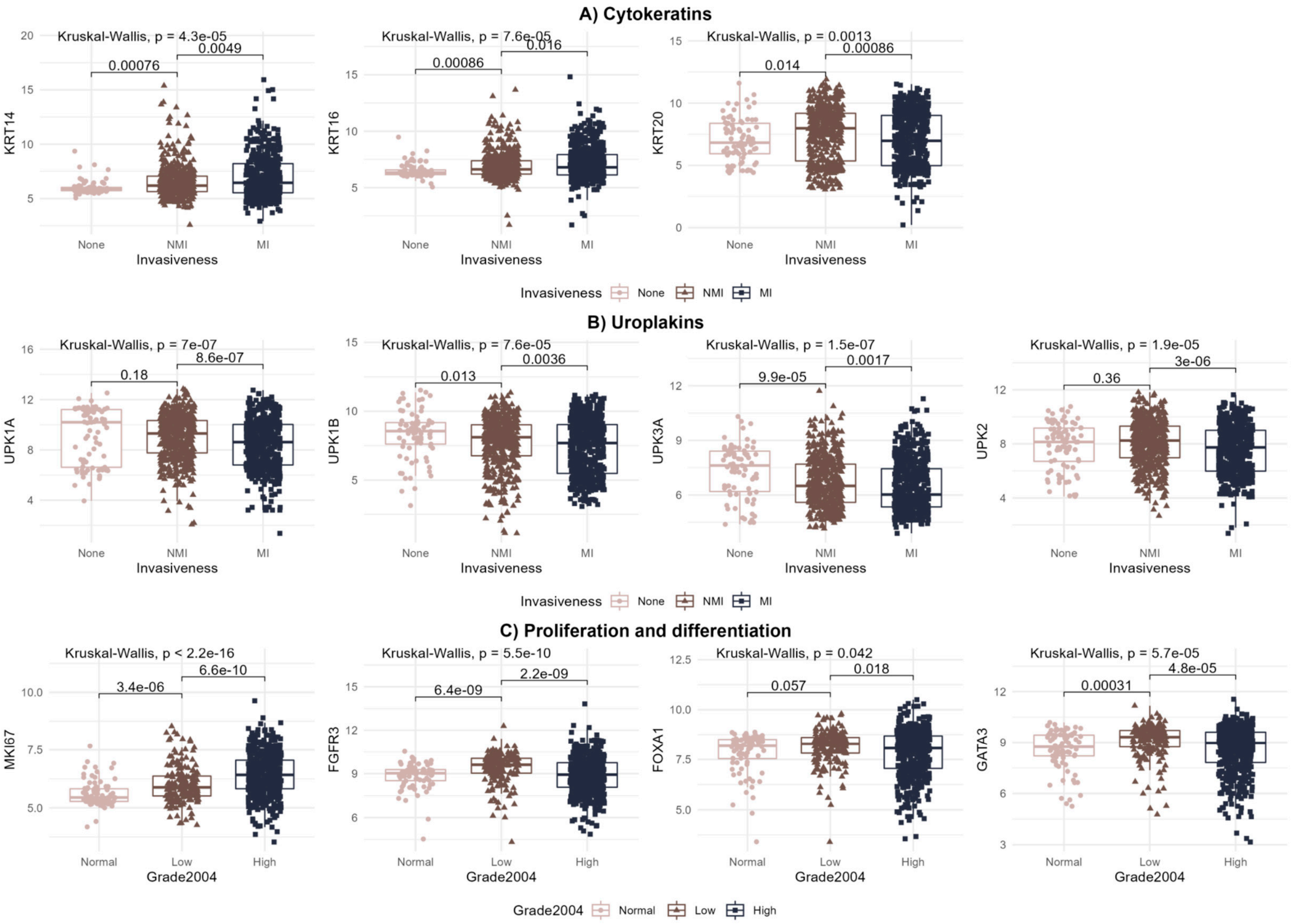

Supplemental Figure S5: Gene expression of 11 known BLCA markers among clinical conditions in the ComBat data

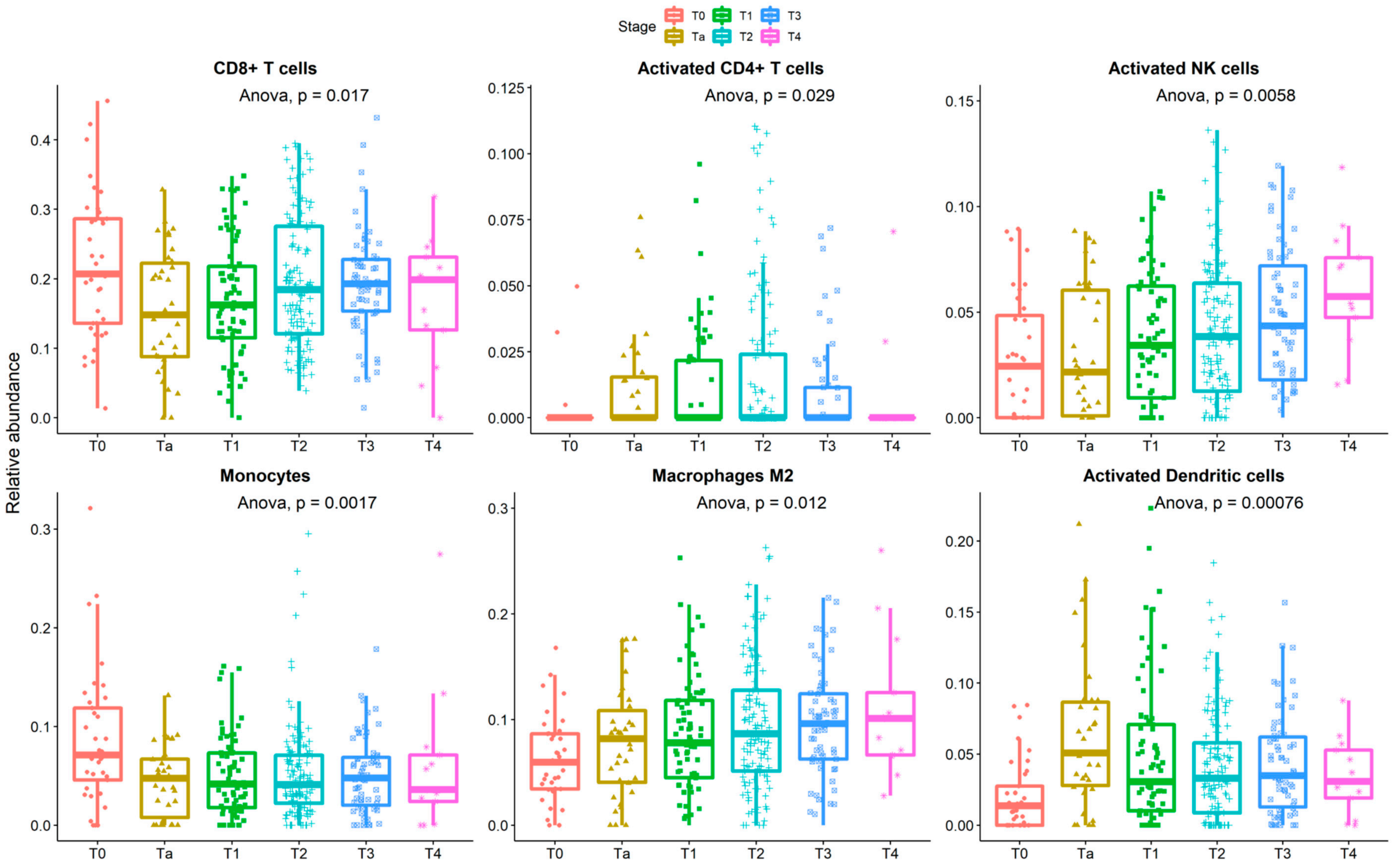

Supplemental Figure S6: Significant results from the CIBERSORT analysis of the ComBat data

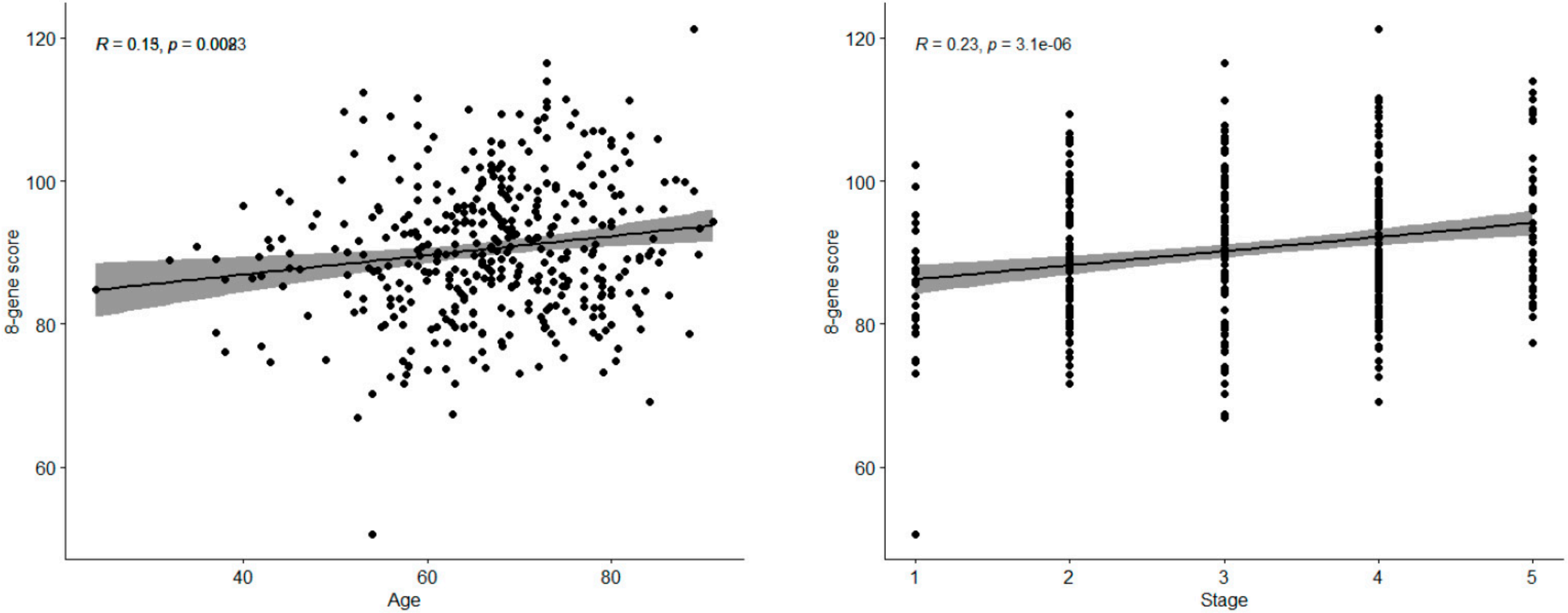

Supplemental Figure S7: Correlation analysis between the 8 gene signature score and the Age (left), and Stage of the patients (right). Stage: 1 = Ta, 2 = T1, 3 = T2, 4 = T3, 5 = T4

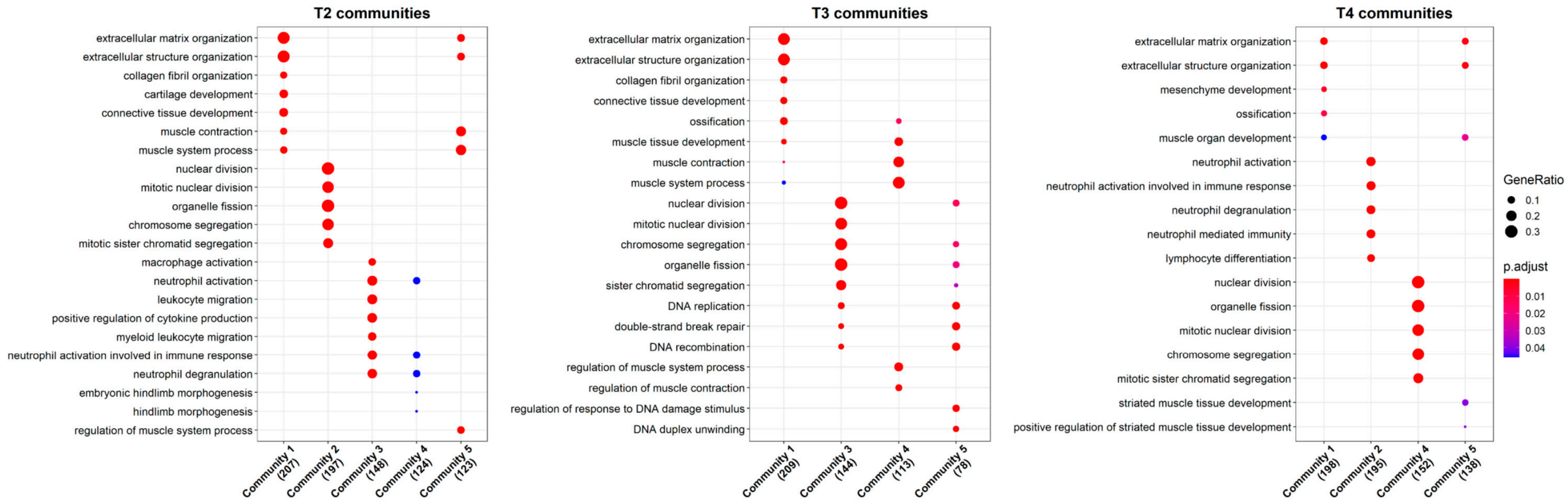

Supplemental Figure S8: Functional annotation of the top 5 in size molecular communities of coexpressed genes detected in the T2, T3 and T4 stages of the TCGA cohort, demonstrating a significant overlap with the cell cycle, extracellular matrix and immunity communities of the discovery data
